# Foot orthoses for the management of low back pain: a qualitative approach capturing the patient’s perspective

**DOI:** 10.1186/1757-1146-6-17

**Published:** 2013-05-07

**Authors:** Anita E Williams, Lindsay A Hill, Christopher J Nester

**Affiliations:** 1 Orthotics and Podiatry, Brian Blatchford Building, University of Salford, Salford, M6 6PU, UK; 2 (Foot & Ankle) Royal Bolton Hospital NHS Foundation Trust, Elective Orthopaedics Service, Bolton, UK; 3 School of Health Sciences, University of Salford, Frederick Road, Salford, M6 6PU, UK

**Keywords:** Low back pain, Foot orthoses, Qualitative research, Information

## Abstract

**Background:**

The onset of non specific low back pain is associated with heavy lifting, age, female gender, and poor general health, with psychological factors being predictors of it becoming chronic. Additionally, it is thought that altered lower limb biomechanics are a contributory factor, with foot orthoses increasingly being considered as an appropriate intervention by physiotherapists and podiatrists. However, research into the effect of foot orthoses is inconclusive, primarily focusing on the biomechanical effect and not the symptomatic relief from the patient’s perspective. The aim of this study was to explore the breadth of patients’ experiences of being provided with foot orthoses and to evaluate any changes in their back pain following this experience.

**Method:**

Following ethical approval, participants (n = 25) with non-specific low back pain associated with altered lower limb biomechanics were provided with customised foot orthoses. At 16 weeks after being provided with the foot orthoses, conversational style interviews were carried out with each patient. An interpretivistic phenomenological approach was adopted for the data collection and analysis.

**Results:**

For these participants, foot orthoses appeared to be effective. However, the main influence on this outcome was the consultation process and a patient focussed approach. The consultation was an opportunity for fostering mutual understanding, with verbal and visual explanation reassuring the patient and this influenced the patient’s beliefs, their engagement with the foot orthoses (physical) and their experience of low back pain (psychological).

**Conclusion:**

Clinicians need to adopt ‘psychologically informed practice’ in relation to the provision of foot orthoses. Likewise, researchers should consider all the influencing factors found in this study, both in relation to their study protocol and the outcomes they plan to measure.

## Background

Non-specific low back pain is one of the most common reasons for people to seek a medical consultation in primary care [1]. Most will improve either naturally or with minimal intervention over the subsequent three months, but some have persistent symptoms which then become chronic [2,3]. Further, up to 70 percent of patients experience a repeat episode within one year of treatment [3,4]. The direct resource costs in the United States are 96 million dollars over a period of 12-months [5], therefore the description of it as a ‘medical disaster’ [6] remains unchanged. Heavy lifting and the accumulation of loads or frequency of lifts are moderate to strong risk factors for the onset of non-specific low back pain, with strong associations for flexed and rotated positions of the lumbar spine [7]. However, abnormal lower limb posture and biomechanics are also associated with its onset and persistence [8].

In order to address the abnormal lower limb biomechanics, it is perceived that the use of foot orthoses has increasingly become a popular choice for clinicians. In part, the rationale for this is supported by Brantingham *et al*. [9] who identified that as a decrease in ankle dorsiflexion, a smaller navicular drop and a high arch are associated with low back pain, foot orthoses should be considered as an intervention. An earlier study by Bird *et al*. [10] suggested that foot orthoses have the potential to influence symptoms as they change the foot and subsequently lower limb movement, influencing muscle activity in the low back area during the gait cycle. This notion is further supported by Zhang [11] who identified that compared with chiropractic care alone, the addition of foot orthoses improved symptoms in standing workers. Further, a small prospective study [12] indicated improvement in low back pain and a recent clinical trial indicated short term benefits with the use of foot orthoses [13].

Guidelines on the management of low back pain [14,15] recommend that initial treatment for low back pain should be the management of pain, exercise therapy, manual therapy, and/or acupuncture. However, despite some indication that foot orthoses may be beneficial [10-13], they have yet to feature in these guidelines as the evidence has been deemed to be inconclusive [16,17].

One issue related to the evidence base for foot orthoses is the scope of outcome measures that have been used. To date, research has taken a narrow perspective of “outcome” focussing on biomechanical [9] and limited physical outcomes [10-13]. However, a more holistic approach to assessing the patient, considering the patients perceptions of the physical, psychological and emotional impact of low back pain may provide a wider perspective on the potential outcomes of foot orthoses as an intervention. This notion is supported by the knowledge that both the psychological and emotional reactions, as described by Greenhalgh and Selfe [18], contribute to the physical manifestations of low back pain. Further, the predictors of persistent symptoms include emotions such as distress, and fear-avoidance beliefs [19]. Therefore, it would seem pertinent to investigate the impact of foot orthoses with the focus being on the psychological and emotional perspectives. Hence, the purpose of this study was to explore the breadth of patients’ experiences of being provided with foot orthoses for the management of their low back pain using a qualitative approach to data collection and analysis.

## Methods

### Participant recruitment

Following ethical approval, (North West 6 -Research Ethics Committee, Greater Manchester South) potential participants were approached by their consultant physiotherapist at a back pain clinic in the North-West of England. The inclusion criteria were that the low back pain had already been investigated and serious pathology (e.g. tumour) excluded; the back pain was associated with an element of biomechanical dysfunction as judged by the consultant physiotherapist; their footwear was able to accommodate foot orthoses and that they had no previous use of foot orthoses. Twenty five participants were recruited in order to capture a wide range of experiences. Written information about the study was provided before informed consent was obtained from each participant.

### Procedure for provision of foot orthoses

A specialist podiatrist carried out a comprehensive clinical assessment of lower limb alignment, foot posture [20] and function. A Roland-Morris Disability Questionnaire [21] was also completed as a standard patient reported outcome measure used in clinical practice. The Gaitscan™ system (Gaitscan™, Toronto, Canada: http://www.theorthoticgroup.com) was used to further inform the clinical findings and to create the design features of the custom polypropylene foot orthoses used. The Gaitscan system translates the timing sequences of the gait cycle and indicates the pressure and centre of pressure during each step into the individual’s requirements for the arch height, rearfoot control and the contours of the foot orthoses. Further, it provides a 3D visual image of the pressures applied to the foot and the overall profile of the foot during the gait cycle. This visual image and the results of the clinical assessment were discussed with the participants in the context of the anticipated effect of the foot orthoses on their lower limb biomechanics. They were then given time to ask questions. When the foot orthoses were fitted, both written and verbal advice was provided in relation to their use. No other treatments were given during this time period. A review was carried out at 16 weeks. At this review appointment, the qualitative data collection was carried out.

### Data collection

Conversational style interviews took place with an interpretive phenomenological approach to the data collection [22]. The interviews were carried out by the specialist podiatrist with the focus of the interviews being the participant’s experience, views and feelings about being provided with foot orthoses for their low back pain. The opening question was used to start the dialogue was “Tell me about your experience of being provided with and wearing orthotics for your low back pain……”

Further trigger questions were used if the participant wandered off the subject for too long. Examples of the trigger questions were, ‘How has your back pain been since wearing your orthoses?’ and ‘What impact have the orthoses had on your ability to do things?’ The questions were kept as open as possible to encourage dialogue with the participant. The interviews were digitally recorded, transcribed verbatim and field notes were used to supplement the data.

### Data analysis

The data analysis employed the steps defined by Colaizzi [23]. All transcripts were read and phrases extracted that pertained to the research focus. The meaning of each phrase was formulated and then these were organised into sub themes. These were then checked against the original descriptions before the final themes were formed and a final global theme defined. The findings were then agreed with the co-authors of this paper and then returned to the participants for verification of the accuracy and interpretation. Morse and Field [24] suggest that this adds to the truthfulness and rigor of the results. The participants verified the findings as being an accurate reflection of their experiences. An iterative approach to the analysis revealed that no new themes emerged after the eighteenth participant. However, the remaining seven responses further illuminated the themes and added to the rigor of the results.

## Results

Fourteen male and 11 female participants with mean age of 44 years (SD = 11.34) had a mean duration of low back pain of 88 months (SD = 13.61). At the review appointment The Roland-Morris Disability Questionnaire mean reduction in total score was 6.46 (SD = 4.34) which is deemed to be clinically significant [21]. However, the focus of this study was the qualitative data which has revealed the wider emotional and psychological factors that influence the participant’s experiences of being provided with the foot orthoses.

Five themes emerged from the data analysis. Exemplars are used from the text to illustrate each theme and each participants name has been replaced with a pseudonym.

### Theme 1: Expectations and understanding

The participants did not anticipate or expect that the feet could be related to symptoms in the low back area. They were surprised to be referred for foot orthoses but as Chris reveals, once they were given an explanation, it gave them hope for resolution of their symptoms:

*“Initially you sort of disbelieve that altering your feet could make any difference to your back, there’s a certain amount of scepticism there…I didn’t think anything could help…no hope until now”* Chris

In particular, if feet had not received attention as a possible cause of their low back pain by other practitioners, such as in the case of Anthony, this had perpetuated their anxiety at not receiving an explanation for the cause and persistence of their symptoms:

“It was a surprise that the back problem was coming from my feet…most of the information had come from ** ***** and he’s a surgeon so he had never looked at my feet…I now feel relieved and am not worrying as I used to” Anthony

Some doubted if the foot orthoses would work at all as they had tried other interventions and failed:

“*I wasn’t expecting that much to be honest…I’d had the back pain that long and nothing else had worked”* Simon

They also had expectations about how long they would take to work with Lisa reporting that initially:

*“I expected them to take a while to work. I thought then…I’m going to keep wearing them because I think it might be a long term thing this…”* Lisa

However, once she had worn them:

*“They had an immediate effect and over time it’s just been better and better. I couldn’t believe it…over a couple of days and I thought …’I like this!’ I am really pleased…I think understanding how my feet were working…or not working… helped enormously”* Lisa

The participant’s expectations and level of knowledge were assessed during the consultation and this appears to have had a positive effect on their beliefs about the link between foot biomechanics and their back problem and hence the potential for the foot orthoses to provide relief for their symptoms. From the practitioners perspective, knowing what experiences (and hence expectations) that the participants had prior to the foot assessment is crucial in being able to provide tailored information and explanation. This appears to be the foundation for achieving the maximum potential symptomatic relief from the foot orthoses. The apparent loss of hope for symptomatic relief and anxiety associated with this appeared to be reduced during their consultation.

### Theme 2: The assessment process

The participants reported that the assessment was thorough compared to previous experiences of assessment of their low back pain:

*“… I thought it was a very thorough assessment and it was good to spend time looking at everything, just to see what was going on, no one had ever spent that long looking before…”* Mark

Also that they had been listened to during the consultation:

“… *don’t know if it’s also because you.ve been listened to that you feel better..I feel valued”* Tina

Field notes revealed that the patients appeared to have anxiety about their back pain and although this was generally not expressed verbally, notes were made of their body language and expression as indicating anxiety. This was mirrored by the relief they expressed when an explanation was given as to the origins of their back pain following the physical examination:

*“…you were the first person to explain to me how my feet were not going down in the same way and that was such a relief… I understood what you were saying and could see it with my own eyes!”* Anthony

They found the Gaitscan™ results illuminating:

*“....I was impressed with the technology of it all and the fact that you could produce an accurate reading of actually what my feet were doing…”* George

…with the visual images in particular aiding their understanding of the relationship between their foot problems and the lower back:

*“…It’s brilliant, yeah, because I can only learn by being shown so unless you show me a picture or how something works then to me it’s just science…”* Tina

The whole assessment process was beneficial in further enhancing their understanding of the relationship between their foot biomechanics and their back pain and the potential for foot orthoses to improve their foot alignment. The time spent explaining to the participants was valued by them and in turn, this made them feel valued. This knowledge appeared to reduce their anxiety and hence positively prepared them for engagement with the foot orthoses.

### Theme 3: The usability of foot orthoses

Despite wearing shoes that were deemed suitable for the provision of foot orthoses, the participant’s identified some issues with them, such as having only one pair and therefore having to swap them around:

*“…I wear them about 60% of the time, as much as I can remember to swap them around…”* Robert

In particular, the women had difficulty fitting them into ‘social’ footwear but this was for short periods of low level activity and hence the requirements for the orthotic intervention were low:

“..*If I go for dinner with a friend I’m not gonna wear them, but whenever I’m at work I’m fine wearing these (trainers), it’s no problem..”* Angela

Indeed, having the choice to wear ‘social’ footwear for periods of low activity may well have positively influenced their use of the foot orthoses in suitable shoes for periods of high activity.

Some found that they had to get used to the foot orthoses but they had received information about this adjustment phase:

*“… They felt quite strange at first....I did notice a few aches in my legs and it did say in the instructions you might experience this as you get used to them…and so I continued… it was helpful to come back for a review …very reassuring”* Mark

The footwear that they needed to wear for different activities has implications for the usability of the foot orthoses. However, they were used the majority of the time and this may be sufficient to maximise the potential for improvements in symptoms. It is clear that supporting information in the form of verbal and written instruction is crucial in relation to their use and continued use, as is a review appointment. All the participants were wearing the foot orthoses at 16 weeks and planned to continue with them.

### Theme 4: The physical experience of using foot orthoses

The participants reported an improvement in posture and symptoms when wearing the foot orthoses:

“…I don’t feel slouchy when I’m walking ......but it really has changed the whole way that I walk. I feel more like my back is straight…” Andrew

They were able to carry out activities better than before:

“…I’m getting up earlier, I’m actually doing something during the day, I’m eating better because I’m exercising and when I go to bed at night I’m sleeping better…” Richard

Additionally, the participants could return to their normal work and hobbies as revealed by Paula:

*“…I can’t imagine I would manage in work without them to be honest, it’s fabulous…”* and Keith,

*“…I wasn’t able to do that part of the job, I.ve been sort of excused that part …I do feel well enough to do that now so I’m happy…”* and James emphasised the impact on his physical activity,

“… Now we try and do 3–4 miles, 5 days a week. Last week we walked Monday, Tuesday, Wednesday, Thursday and Friday…”

The participants’ experiences of reduced pain and their awareness of improvements in gait, balance and posture has been a positive outcome in that they are able to increase activities that had previously been limited. Their awareness gait, balance and posture could be based on the initial explanation provided following the biomechanical assessment and their knowledge about how foot orthoses work. Also the short term benefits associated with the foot orthoses encourage their continued use of them and improves the likelihood of sustained benefits.

### Theme 5: The psychological experience of using foot orthoses

The participants reported that they experienced an increase in their confidence in moving around when wearing their foot orthoses. Associated with this was a reduction in the fear that they had about the back pain:

“… That fear, the dread! Ive managed to dispel, I’ve had pain off and on, off and on, and initially you think it is coming back but as the weeks and the months go by I don’t think like that…” James

Some expressed a change in attitude to life and that they were hopeful for their future as Anthony expresses:

*“…It’s about 50% better now so I’m hopeful for the future…”* and Paula reveals that *“…wearing the orthotics have helped me to get on with it, my whole attitude has changed because I’m not in pain…”*

Further, Fiona reported a positive effect on her relationships:

“....my husband even said to me the other day” – “when is it you are going back? It’s been better hasn’t it? I thought so because you’ve not been moaning…”

The reduction in pain has an effect on aspects of their lives which in turn has had a positive effect on their mental and emotional wellbeing. It could be perceived that this has had a further effect on their experience and hence the impact of symptoms. This appears to be a cyclical effect of pain reduction, improvement in function and activities, improvement in and hence pain reduction.

These five themes are merged into one global theme which is the main message based on the findings from these participants.

### Global theme

Foot orthoses (physical component) can be successful in the management of people with low back pain if their expectations and information needs are met (psycho-social component) which in turn can lead to a change in their preconceived beliefs in this area.

## Discussion

This qualitative study has provided new insight into how the process of providing foot orthoses contributes to the success in achieving improvements in the participant’s experience of low back pain. The interviews revealed that for these participants, the foot orthoses did improve back pain. This result is supported with the results of the Roland-Morris Disability Questionnaire [21] which was completed as a standard ‘clinical’ outcome measure.

The most revealing aspect of this work is that the physical effects of the foot orthoses is clearly augmented by the defined process of providing them which considers the psychological, emotional and social factors. As it has previously been identified that psychological reactions contribute to low back pain and its persistence [18,19], it would seem that to address these factors in the assessment process is essential if the positive physical effect of the foot orthoses is to be maximised.

When considering how the process of providing the foot orthoses has impacted on the participants experiences, it became clear that there were differences between the ‘research’ assessment process and the usual ‘clinical’ assessment. The podiatrist as the researcher in this study had twice the amount of time with each of the participants than in the usual clinical context. Further, the process of informed consent included a patient information sheet, verbal explanation and opportunity for the participants to ask questions. Their suitability for inclusion into the study involved the researcher asking the potential participants about their understanding of how their back pain could be related to lower limb function, information as to how they would be assessed and then how foot orthoses effect changes in lower limb mechanics. In addition to the usual assessment of the structure and function of the lower limb, part of the assessment process involved producing a 3D image of the participant’s feet showing the results of abnormal lower limb function. This was shown to the participants with verbal explanation as to the meaning of the image. The interplay of all these factors may have contributed to the reported feelings of being valued and hence their anxieties reduced.

Anxiety and other psychological effects have not been measured in studies that have investigated foot orthoses as an intervention [9-13] with the focus being the physical effects. Main and George [25] suggested the notion of 'psychologically informed practice', which they describe as the integration of physical and psychological approaches to treatment of low back pain. It has been highlighted by Main *et al*. [26] that in addition to the physical effects of interventions, attention needs to be given to the beliefs/expectations, emotional responses and behavioural responses associated with low back pain, as these are associated with outcome of treatment. Overmeer *et al*. [27] provide an important model for future studies in this area and clearly recommend evaluation of the psychological effects of living with pain as an influence on the patient’s experience and on the outcome of interventions.

From the results of this study, it is clear that a consultation style in which the clinician spends time listening to these patients’ expectations, beliefs and fears, with explanation about the potential reasons for their back pain, can have the effect of reducing their anxiety and hence their experience of pain. It is known that anxiety can influence the level of pain experienced by individuals [28] and it might also become a barrier to symptom improvement through its negative effect on compliance. Good communication skills that are underpinned by a psychosocial approach are the basis for an effective consultation and also to patient compliance with interventions [29]. Such is the potential impact of communication on outcomes, that allowing sufficient time for the consultation and effective communication could be seen as part of the intervention, rather than simply the process through which the intervention is dispensed. Indeed, communication skills have been identified as worthy of investigation in relation to physiotherapy consultations with patients with low back pain [30] and we suggest that this should also be investigated in relation to the provision of foot orthoses.

The use of technology to visually explain the patients underlying problem with foot function and how foot orthoses work can improve engagement and might alter expectations too. This reinforces the vital link between the problem and the potential cure. The visual component of gait analysis has been shown to be beneficial in demonstrating the effects of footwear in a cohort of elderly people [31]. Vilallonga [32] identified that imagery improves communication between patients and clinicians and hence improves understanding, compliance and symptom improvement. The participants in this study valued the time that was spent with them in relation to listening, and visual and verbal explanation and this clearly had an effect on reducing their anxiety.

One of the challenges in this study was that the foot orthoses had to be worn in footwear that can accommodate them. It is known that this can be an obstacle [33] and it is clear that careful explanation about footwear choices in relation to levels of activity is crucial in maintaining the choices that people expect in their everyday lives. That the foot orthoses were not worn for social but low impact activities may not be a problem in relation to outcomes, since these occasions are typically of short duration. Indeed it can be said that maintaining choice is important in relation to patient compliance over the period of time required to achieve and maintain improvements.

A further influence on their engagement with foot orthoses could be that the participants were reassessed after 16 weeks. This provided the opportunity for reinforcement of information and an evaluation of the short term outcomes such as improved posture. This could have encouraged the participants to continue with their use and may also have elevated their tolerance of any issues, such as the inconvenience of swapping orthoses between footwear, or having to change their footwear styles. It is therefore of concern that there is anecdotal evidence that in some health care settings reassessment and review of foot orthoses is not routine practice due to increasing demands for shorter waiting lists and limited appointments.

Potential limitations to this study could be that a heterogeneous population was utilised in relation to age range and duration of low back pain. However, the aim of the research was to explore experiences of these participants and in this respect the purposive sampling has provided results which illuminate influences on an area of practice which has previously been ignored. Furthermore, the research setting was a pain clinic focusing on chronic low back pain cases that had failed to respond to other treatments and thus the sample represents the reality of practice for this particular setting.

In respect of future research, both Sahar *et al*. [16] and van Middelkoop *et al*. [17] recommended large scale clinical trials of foot orthoses. However, as this study has demonstrated, there are complex patient focussed factors which have the potential to influence the outcome. These factors are the patients understanding about the cause of their low back pain, their level of anxiety, expectations about the foot orthoses as an intervention, their initial experience of wearing them and the process by which they are provided. These factors may be independent or complementary to the physical effect of the foot orthoses. Either way, we consider that it is crucial that these factors are considered during the planning of future clinical trials. The most recent clinical trial [13] demonstrated that the physical effect of foot orthoses achieved short term benefits. However, they did not detail any of the factors that may have contributed to the short term successful outcome or may influence sustained success.

If these multiple influences on outcome are ignored, there is the risk incomplete appraisal of the value and role of foot orthoses for whatever condition that they are provided for. This seems especially critical in the context of health policy that continually elevates the importance of the voice of patients in relation to their own care [34]. Arguably, all of the factors identified in this study could all be reasonably considered as part of the intervention. This would move foot orthoses from being a mechanical to a “complex intervention” [35] and in turn could influence both the future research approach and the approach to their provision in clinical practice.

## Conclusion

The authors conclude that clinicians need to adopt 'psychologically informed practice' in relation to the provision of foot orthoses. Likewise, researchers should consider all the influencing factors found in this study both in relation to their study protocol and the outcomes they plan to measure.

## Competing interests

'The authors declare that they have no competing interests.

## Authors’ contributions

AW – conceived the idea for a qualitative approach – co-analysed the data and created the paper; LH - carried out the data collection and the primary data analysis – contributed to the presentation of the results in the paper; CN- co-analysed the data and contributed to the paper. All authors read and approved the final manuscript.
